# Rival phytoplankton contribute to the cross protection of *Prochlorococcus* from oxidative stress

**DOI:** 10.1128/aem.01128-24

**Published:** 2025-04-10

**Authors:** Benjamin C. Calfee, Emily C. Bowden, Erik R. Zinser

**Affiliations:** 1 Department of Microbiology, University of Tennessee 189504 , Knoxville, Tennessee, USA; Washington University in St. Louis, St. Louis, Missouri, USA

**Keywords:** hydrogen peroxide, *Prochlorococcus*, *Synechococcus*, picoeukaryotes, black queen hypothesis

## Abstract

The marine cyanobacterium *Prochlorococcus* numerically dominates the phytoplankton communities in all lower latitude, open ocean environments. Having lost the catalase gene, *Prochlorococcus* is highly susceptible to exogenous hydrogen peroxide (H_2_O_2_) produced at the ocean’s surface. Protection by H_2_O_2_-scavenging heterotrophic “helper” bacteria has been demonstrated in laboratory cultures and implicated as an important mechanism of *Prochlorococcus* survival in the ocean. Importantly, some other phytoplankton can also scavenge H_2_O_2_, suggesting these competing microbes may inadvertently protect *Prochlorococcus*. In this study, we assessed the ability of co-occurring phytoplankton, the cyanobacterium *Synechococcus* and picoeukaryotes *Micromonas* and *Ostreococcus*, to protect *Prochlorococcus* from H_2_O_2_ exposure when cocultured at ecologically relevant abundances. All three genera could significantly degrade H_2_O_2_ and diminish *Prochlorococcus* mortality during H_2_O_2_ exposures simulating photochemical production and rainfall events. We suggest that these phytoplankton groups contribute significantly to the H_2_O_2_ microbial sink of the open ocean, thus complicating their relationships with and perhaps contributing to the evolutionary history of *Prochlorococcus*.

**IMPORTANCE:**

The marine cyanobacterium *Prochlorococcus* is the most abundant photosynthetic organism on the planet and is crucially involved in microbial community dynamics and biogeochemical cycling in most tropical and subtropical ocean waters. This success is due, in part, to the detoxification of the reactive oxygen species hydrogen peroxide (H_2_O_2_) performed by “helper” organisms. Earlier work identified heterotrophic bacteria as helpers, and here, we demonstrate that rival cyanobacteria and picoeukaryotic phytoplankton can also contribute to the survival of *Prochlorococcus* during exposure to H_2_O_2_. Whereas heterotrophic bacteria helper organisms can benefit directly from promoting the survival of carbon-fixing *Prochlorococcus* cells, phytoplankton helpers may suffer a twofold injury: production of H_2_O_2_ degrading enzymes constrains already limited resources in oligotrophic environments, and the activity of these enzymes bolsters the abundance of their numerically dominant competitor. These findings build toward a better understanding of the intricate dynamics and interactions that shape microbial community structure in the open ocean.

## INTRODUCTION

Organisms within sunlit aquatic environments are exposed to reactive oxygen species (ROS) generated biotically as metabolic by-products (1–7) and abiotically through photooxidation of organic substances (8–12) and from rainfall (13–16). Hydrogen peroxide (H_2_O_2_) makes up a significant proportion of total ROS in aquatic environments and is consistently detected in surface mixed layers across oceanic basins (17–19). Biotic production of H_2_O_2_ varies drastically, as organisms within the same trophic level can serve as sinks or sources due to differences in metabolism and physiology (1, 2, 6, 20). Total daily abiotic production at the surface layer of the oligotrophic ocean has been estimated at approximately 800 nM, but the microbial sink—a community-wide collection of catalases, peroxidases, and other H_2_O_2_-degrading molecules—maintain concentrations below 200 nM (21–24). While exposure to high concentrations of H_2_O_2_ is lethal to organisms lacking these enzymes, even those that possess them can experience cellular damage (25–27) and alterations in physiological processes (28–30).

Cyanobacteria of the genus *Prochlorococcus* thrive in sunlit open ocean environments and are numerically dominant over other phytoplankton such as cyanobacteria of the *Synechococcus* species and small (<2 µm) eukaryotic phytoplankton, *Micromonas* and *Ostreococcus* (31–34). The numerical dominance of *Prochlorococcus* is often attributed to their streamlined genome and small cell size that provides a higher growth efficiency at the cost of diminished stress response and fewer DNA repair mechanisms (35–38), among other losses in physiological capability.

Due to the evolutionary loss of a functioning catalase enzyme, *Prochlorococcus* are dependent upon other members of the microbial community for the detoxification of ROS—specifically hydrogen peroxide (H_2_O_2_)—in the surface mixed layer (22, 39). Prior research demonstrated heterotrophic “helper” bacteria can remove H_2_O_2_ from the medium and provide significant protection to *Prochlorococcus* against this reactive oxygen species (40, 41). One such helper is the heterotrophic bacterium *Alteromonas macleodii,* which is often co-isolated alongside *Prochlorococcus* and has been shown to both provide efficient protection against H_2_O_2_ and cause significant changes in gene expression when in coculture with *Prochlorococcus* (22, 40, 42–45).

Analysis of metatranscriptomes from the sun-exposed surface of the open ocean revealed transcripts of both heterotroph and phytoplankton genes involved in the degradation of hydrogen peroxide (23). We hypothesized that while less abundant than the heterotrophic community (34, 46, 47), the cyanobacterium *Synechococcus* and photosynthetic picoeukaryotes including the prasinophytes *Ostreococcus* and *Micromonas* could contribute to the microbial sink of hydrogen peroxide. Indeed, *Synechococcus* and other phytoplankton show significant potential to degrade hydrogen peroxide in culture (24, 48–50).

To address this possibility, we co-cultured *Prochlorococcus* with strains of *Synechococcus*, *Micromonas*, and *Ostreococcus* to ascertain whether these co-occurring phytoplankton could help *Prochlorococcus* survive H_2_O_2_ exposures typical of the open ocean. We observed that all strains had significant H_2_O_2_ degradation rates and could improve *Prochlorococcus* survival during both rapid and gradual increases in H_2_O_2_ that cells can experience during rainfall events and daily solar exposures, respectively.

## RESULTS

### Monoculture response to H_2_O_2_

**Fig 1:**
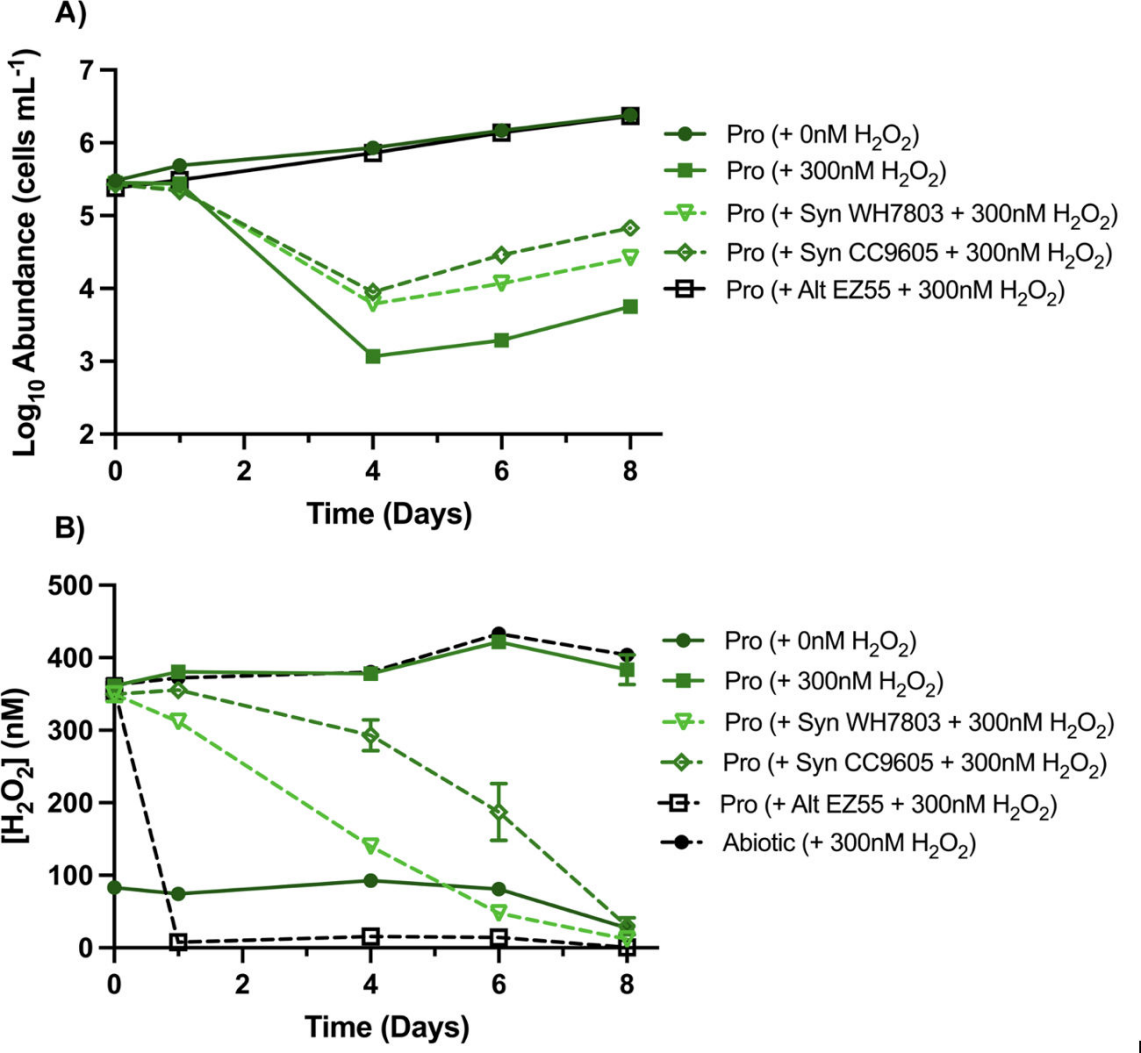
Survival of *Prochlorococcus* during simulated rainfall. (**A**) Growth of *Prochlorococcus* strain MIT9215 (abbreviated Pro in figure key) in mono- and coculture with *Synechococcus* (strains WH7803 or CC9605) or *Alteromonas macleodii* strain EZ55, (co-) inoculated into AMP-PE artificial seawater medium containing <100 nM (unamended) or 350 nM H_2_O_2_. (**B**) Concentrations of H_2_O_2_ in these treatments and an abiotic control over the course of the experiment (*n* = 2). In the legend for this and subsequent figures, information within the parentheses describes addition(s) of a particular strain/treatment. For this and all subsequent figures, the standard deviation of the mean (for H_2_O_2_) or geometric mean (for cells) is shown as error bars for every sample point; for very low standard deviations, error bars are too small to be seen behind the symbols.

Growth of *Prochlorococcus* strain MIT9215 was unimpeded by low extracellular concentrations (<100 nM) of H_2_O_2_ that typify most exposures in the open ocean surface (Fig. 1A) (22, 51). This unimpeded growth occurred despite the inability of this strain to deplete extracellular H_2_O_2_ unless the population exceeds ecologically relevant concentrations (>10^6^ cells mL^−1^). In contrast, exposure to a simulated rainfall addition of 350 nM H_2_O_2_ resulted in a rapid 100-fold drop in cell counts (Fig. 1A). Notably, despite an inability to degrade the H_2_O_2_ (Fig. 1B), the surviving ~1% of the population resumed growth by day 6 (Fig. 1A). H_2_O_2_ concentrations in abiotic controls changed little over the course of the experiment, suggesting that abiotic production and degradation of H_2_O_2_ were negligible (Fig. 1B; S1C and D).

In contrast to *Prochlorococcus*, catalase-positive strains of *Synechococcus* were unaffected by the simulated rainfall conditions of 350 nM H_2_O_2_ (Fig. S1A and B). Strains WH7803 and CC9605 inoculated at ecologically relevant concentrations [~10^4^ cells ml^−1^, (52)] grew at the same rate whether initial H_2_O_2_ concentrations were below 100 nM or at 350 nM. Like *Prochlorococcus*, both strains decreased H_2_O_2_ concentrations when the initial H_2_O_2_ concentration was below 100 nM, but only after several days of growth. In contrast, when exposed to the higher concentration (350 nM), both *Synechococcus* strains degraded H_2_O_2_ throughout their growth, depleting it to near or below the limit of detection (~10 nM) by day 8 (Fig. S1C and D).

Like the *Synechococcus* strains, the growth of the picoeukaryotic phytoplankton *Micromonas commoda* strain RCC299, *Micromonas pusilla* strain CCMP 1545, and *Ostreococcus lucimarinus* strain CCMP2972A was unaffected by 350 nM H_2_O_2_ (Fig. S2A through C). Under the high H_2_O_2_ conditions, picoeukaryotes degraded H_2_O_2_ concentrations at a similar rate (Fig. S2E) but had no impact on H_2_O_2_ at low (<100 nM) concentrations (Fig. S2D).

### Cross-protection from rapid increases in H_2_O_2_: rainfall simulation

To assess the potential for catalase-positive strains of *Synechococcus* to protect *Prochlorococcus* from oxidative damage, co-cultures were inoculated in AMP-PE medium amended with 300 nM H_2_O_2_ (350 nM total). The growth (Fig. S1A and B) and peroxide scavenging rates (Fig. 1B) of the two *Synechococcus* strains were unaffected by the presence of *Prochlorococcus*. Critically, the mortality of *Prochlorococcus* caused by the simulated rainfall H_2_O_2_ exposure at 350 nM was reduced by an order of magnitude when cocultured with ecologically relevant concentrations of either *Synechococcus* WH7803 (*P* = 0.0007) or CC9605 (*P* < 0.0005) (Fig. 1A). By comparison, coculture of *Prochlorococcus* with the heterotroph *Alteromonas macleodii* strain EZ55 mitigated all negative effects of H_2_O_2_ exposure by decreasing concentrations below the level of detection by day one (Fig. 1A).

**Fig 2:**
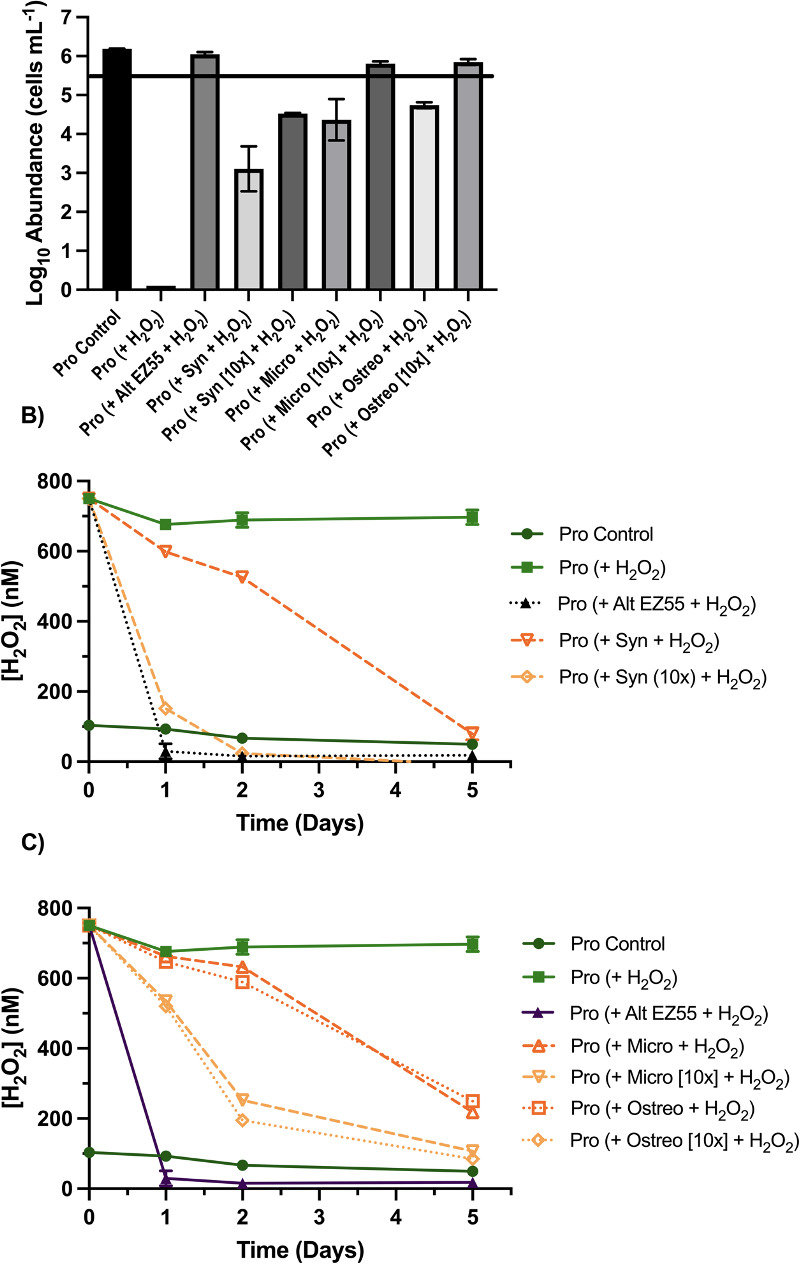
Effect of instantaneous H_2_O_2_ addition and increased helper abundance. (**A**) Final abundances (day 5) of *Prochlorococcus* strain MIT9215 (abbreviated Pro in figure key) in mono- and coculture with *Synechococcus* strain WH7803, *Micromonas commoda* strain RCC299, *Ostreococcus lucimarinus* strain CCMP2972A, or *Alteromonas macleodii* strain EZ55 in AMP-PE artificial seawater medium exposed to an instantaneous addition of ~750 nM H_2_O_2_. The initial abundance of photosynthetic helpers was either 1× or 10× ([10×]) their ecologically relevant abundance. The initial abundance of *Prochlorococcus* (10^5^ cells mL^−1^) is depicted by a horizontal black line. Concentrations of H_2_O_2_ were quantified over the course of the experiment for cocultures with (**B**) *Synechococcus* and (**C**) picoeukaryotic phytoplankton (*n* = 2).

**Fig 3:**
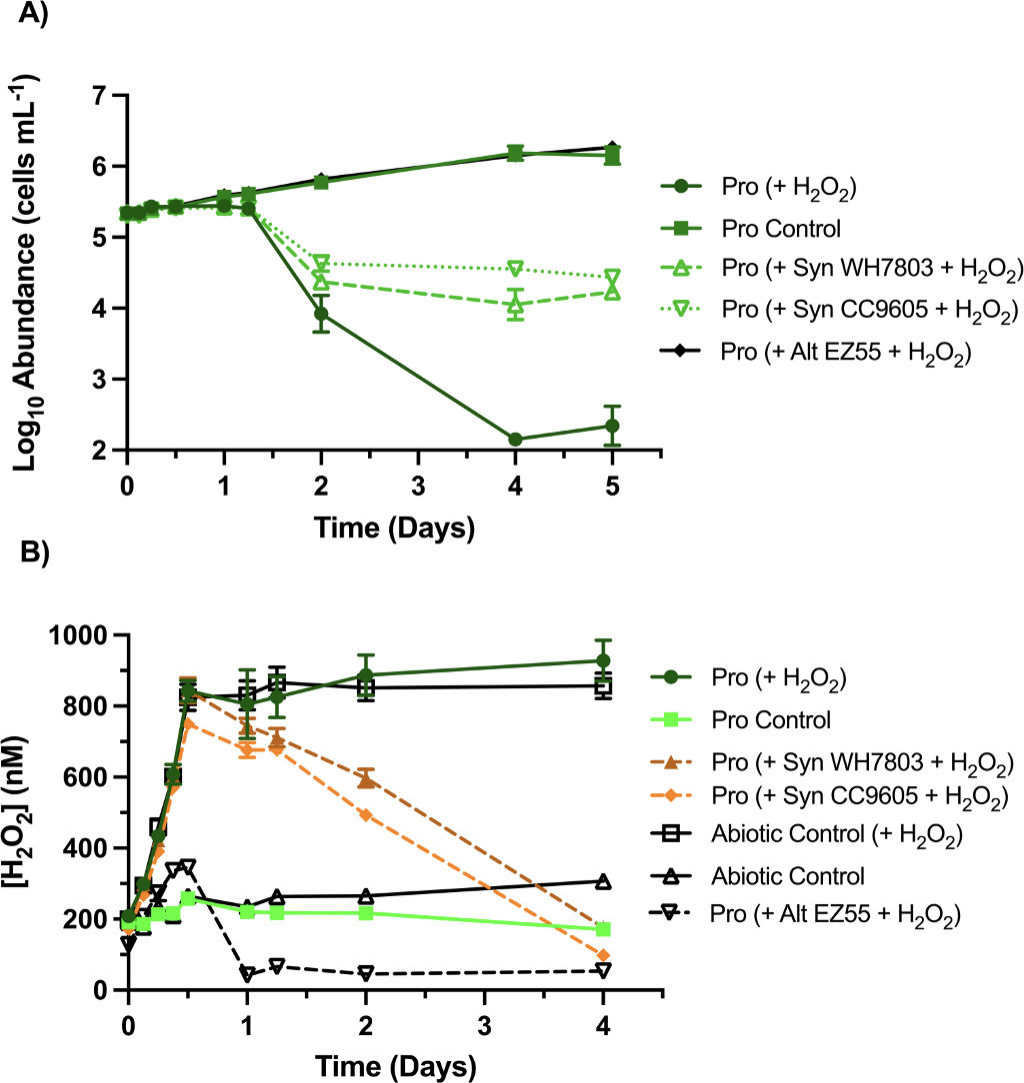
Survival of *Prochlorococcus* with *Synechococcus* after simulated photochemical production of H_2_O_2_. (**A**) Growth of *Prochlorococcus* strain MIT9215 (abbreviated Pro in figure key) in mono- and coculture with *Synechococcus* (strains WH7803 or CC9605) or *Alteromonas macleodii* strain EZ55, (co-)inoculated into AMP-PE artificial seawater medium provided with an incremental addition of 800 nM H_2_O_2_ over the course of the daylight portion of a single diel. (**B**) Concentrations of H_2_O_2_ in these treatments and an abiotic control over the course of the experiment (*n* = 3).

In prior studies, we discovered heterotroph helper EZ55 can protect *Prochlorococcus* when both are inoculated into medium containing high H_2_O_2_ concentration (800 nM) (22). We confirmed that in this study as well (though H_2_O_2_ concentrations after supplementation were closer to 750 nM) (Fig. 2A and 3). H_2_O_2_ in these mixed cultures was completely degraded within the first day of the experiment (Fig. 2B and C). By comparison, we observed that *Synechococcus* and picoeukaryotes can likewise protect *Prochlorococcus* from this high exposure, but not as well as EZ55. While *Synechococcus* strain WH7803 (Fig. S3A) or picoeukaryotes *Micromonas commoda* strain RCC299 or *Ostreococcus lucimarinus* strain CCMP2972A (Fig. S3B and C) co-inoculated at ecologically relevant abundances did not prevent the initial 100-fold decline of *Prochlorococcus* seen in monocultures, they did prevent any further mortality, with *Prochlorococcus* abundances at day 5 of about 1,000 cell mL^−1^ with *Synechococcus* (*P* < 0.0001) and 10,000 cell mL^−1^ with picoeukaryotes (*P* < 0.0001) (Fig. 2A).

To determine the influence of initial helper abundance on H_2_O_2_ degradation and protection, we repeated these coculture experiments having a 10-fold increase in starting inoculum of either *Synechococcus* (10^5^ cells mL^−1^) or picoeukaryotes (10^4^ cells mL^−1^), concentrations not commonly observed in the open ocean but useful to assess density dependence. Increasing cell concentration dramatically increased the rate of peroxide degradation, particularly for *Synechococcus* (Fig. 2B and C and Table S1). As a result of greater helper abundance, final coculture abundances of *Prochlorococcus* on day 5 of the experiment were roughly 10-fold greater (Fig. 2A). Heat-killed cells at similar concentrations showed no protective effect for *Prochlorococcus*, and except for the marginal effect of the 10-fold higher *Micromonas* cell addition, showed no ability to lower H_2_O_2_ concentrations relative to the no cell addition control (Fig. S4).

### Cross-protection from gradual increases in H_2_O_2_: photochemical simulation

To address how *Prochlorococcus* and potential helper organisms respond to a dynamic rather than static source, we provided H_2_O_2_ to the medium in stepwise additions during the experimental daytime of the first day of incubation (Fig. 3B). Monocultures of *Prochlorococcus* strain MIT9215 exposed to this H_2_O_2_ regime showed over 99.9% mortality and reached their lowest abundance by day 4 (Fig. 3A). Coculturing with *Synechococcus* strains WH7803 or CC9605 limited the mortality of *Prochlorococcus* to one order of magnitude (*P* = 0.0003 and 0.0002, respectively) (Fig. 3A), with H_2_O_2_ decreasing to a sublethal concentration by day 4 (Fig. 3B). These *Synechococcus* strains, themselves unaffected by the elevated exposure (Fig. S5A), made marginal impacts on H_2_O_2_ during the incremental additions but subsequently depleted the H_2_O_2_ over the days that followed (Fig. 3B). H_2_O_2_ mortality was completely prevented when *Prochlorococcus* was cocultured with EZ55, and final abundances matched the monoculture controls without H_2_O_2_ additions (Fig. 3A). Whereas the cyanobacteria provided marginal H_2_O_2_ degradation by day 1, EZ55 caused substantial degradation within the first 12 hours, total degradation by the end of the first day, and always maintained concentrations below 400 nM (Fig. 3B).

**Fig 4:**
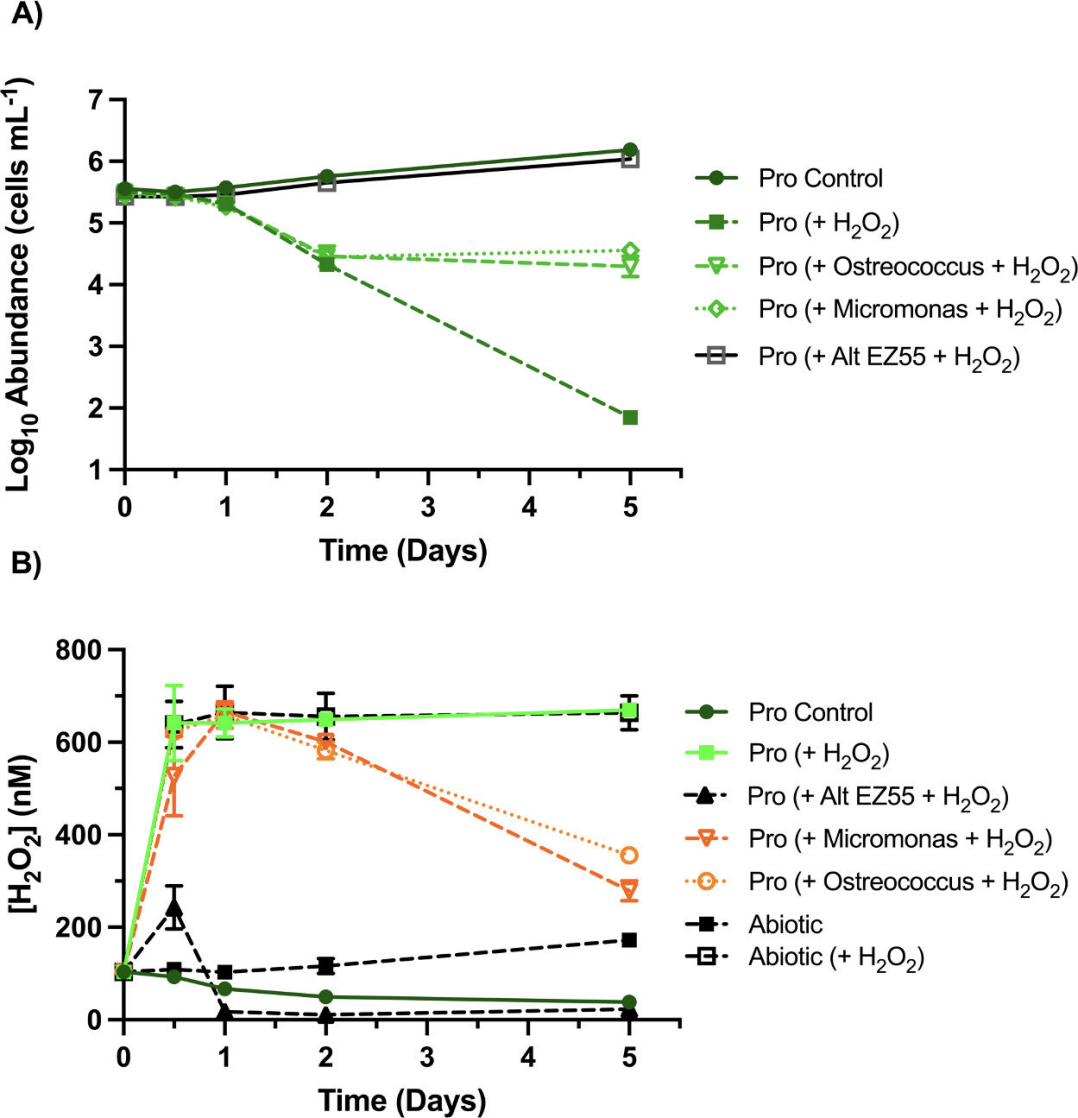
Survival of *Prochlorococcus* with picoeukaryotes after simulated photochemical production of H_2_O_2_. (**A**) Growth of *Prochlorococcus* strain MIT9215 (abbreviated Pro in figure key) in mono- and coculture with *Micromonas commoda* strain RCC299, *Ostreococcus lucimarinus* strain CCMP2972A, or *Alteromonas macleodii* strain EZ55, (co-)inoculated into AMP-PE artificial seawater medium provided with an incremental addition of 650 nM H_2_O_2_ over the course of the daylight portion of a single diel. (**B**) Concentrations of H_2_O_2_ in these treatments and an abiotic control over the course of the experiment (*n* = 2).

Like *Synechococcus*, growth of picoeukaryotes *M. commoda* strain RCC299 and *O. lucimarinus* strain CCMP2972A was unaffected by the H_2_O_2_ additions (Fig. S4B) and coculture limited *Prochlorococcus* mortality to one order of magnitude (*P* < 0.0001) (Fig. 4A) even though picoeukaryote abundance was an order of magnitude less than *Synechococcus* (Fig. S5). This equivalent outcome was surprising, however, as H_2_O_2_ degradation was slower than cocultures with *Synechococcus*, ultimately exposing *Prochlorococcus* to higher concentrations of H_2_O_2_ over the course of the 5-day experiment (Fig. 3B and 4B).

### Exposure time influence on mortality

**Fig 5:**
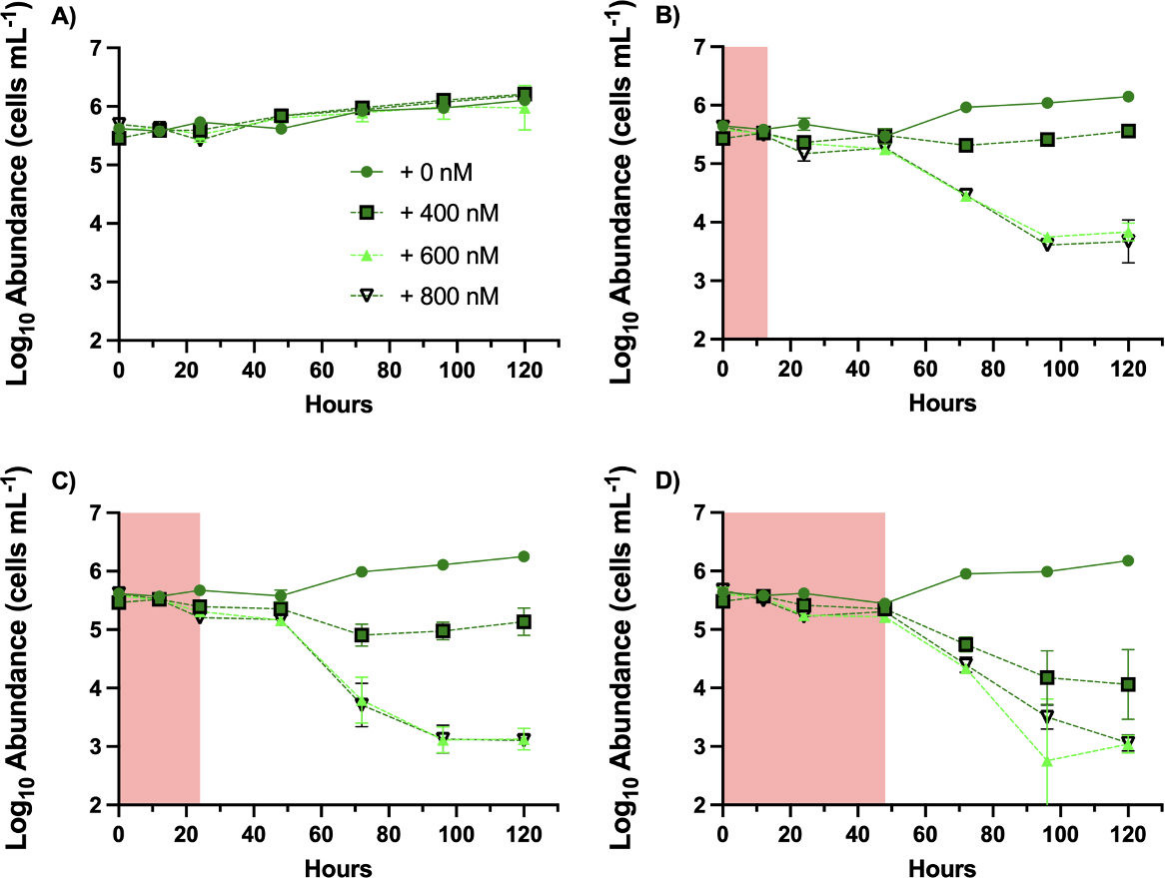
*Prochlorococcus* survival during and after varying exposures to H_2_O_2_. Growth of *Prochlorococcus* strain MIT9215 in AMP-PE artificial seawater medium supplemented with 0, 400, 600, or 800 nM H_2_O_2_. H_2_O_2_ was eliminated after (**A**) 0, (**B**) 12, (**C**) 24, or (**D**) 48 hours of exposure by the addition of 500 μM sodium pyruvate. Pink shading indicates the period of exposure to H_2_O_2_ (*n* = 3).

Survival and recovery after acute exposures have not been tested but could inform the results of the “rainfall” simulations and exposures to highly dynamic H_2_O_2_ exposures in the presence of actively detoxifying helpers. *Prochlorococcus* strain MIT9215 was inoculated into AMP-PE medium supplemented with 0, 400, 600, or 800 nM H_2_O_2_ (Fig. 5; Fig. S6). After 0-, 12-, 24-, or 48 hours sodium pyruvate was added to completely eliminate H_2_O_2_ for the remainder of the 120 hour incubation. While cultures without H_2_O_2_ amendment or with immediate depletion of H_2_O_2_ by sodium pyruvate exhibited growth after 48 hours, all other conditions began to show mortality by 48 hours post inoculation (Fig. 5). Exposure to 400 nM H_2_O_2_ for 12, 24, or 48 hours resulted in surviving populations of 60%, 30%, and 6% of the starting inoculum, respectively. Exposure to either 600 or 800 nM H_2_O_2_ for 12 hours or longer caused 99% mortality in *Prochlorococcus* populations (Fig. S6).

When extrapolated to the previous coculture experiments, these data suggest that H_2_O_2_ concentrations experienced by *Prochlorococcus* in the first days of coculture during instantaneous or incremental additions were sufficient to later reduce *Prochlorococcus* from 10^5^ to ≤10^3^ cells mL^−1^.

## DISCUSSION

The microbial community serves as the primary sink for hydrogen peroxide in the sunlit ocean (21, 22, 24), and in this study, we demonstrated that photosynthetic microbes at ecologically relevant concentrations can contribute to this sink and confer protection to *Prochlorococcus*. This work extends prior studies demonstrating the protective capacity by catalase-positive heterotrophic bacteria (22, 41) and provides direct evidence of protection by phytoplankton as suggested from molecular field observations (22, 23, 40) and from degradation kinetics from monoculture studies (20, 24, 50). Here, we showed evidence that cooccurring cyanobacteria (*Synechococcus*) and picoeukaryotic phytoplankton (*Micromonas* and *Ostreococcus*) can protect *Prochlorococcus* when cocultured at environmentally observed abundances though clearly none of the phytoplankton strains alone could offer complete protection. Protection by these phytoplankton types appears to be an active (e.g., enzymatic) process, as heat-killed cells lacked the protective effect of the live cells, though an excessive abundance of dead picoeukaryotes did show some ability to remove peroxide from the medium.

Importantly, protection of *Prochlorococcus* by *Synechococcus* and the picoeukaryotes occurred under both instantaneous and incremental H_2_O_2_ increases that simulated rainfall and photochemical production sources, respectively, that operate in the open ocean (13–16, 22). Growth of *Prochlorococcus* strain MIT9215 was unimpeded by low extracellular concentrations (<100 nM) of H_2_O_2_ that typify most exposures in the open ocean surface (22, 51). In contrast, exposures to either 350 nM H_2_O_2_—a concentration observed in the open ocean surface mixed layer during rainfall events (13, 14, 16)—or ~800 nM (mean daily photochemical production at the surface) (23) resulted in rapid drops in *Prochlorococcus* cell counts. Under these conditions, co-cultured *Synechococcus* or picoeukaryotes at ecologically relevant concentrations degraded the exogenous H_2_O_2_ and lessened the death of *Prochlorococcus*. By comparison, the heterotroph *Alteromonas* strain EZ55 rapidly degraded the peroxide and *Prochlorococcus* growth was unimpeded relative to a no-peroxide-addition control. As we discuss later, the better protection by the heterotroph owes some of its basis in the higher abundance of the organism in co-culture.

At the surface of the open ocean, photochemical production of H_2_O_2_ occurs gradually over the course of the day (17, 19, 23, 53, 54), and we simulated this in laboratory cultures by stepwise additions over the course of the first day to arrive at 800 nM. Whereas degradation by the heterotroph *Alteromonas* strain EZ55 could keep up with production on day 1, the *Synechococcus* and picoeukaryotes made only marginal contributions to H_2_O_2_ decay in the first day though they did degrade the peroxide over the next several days. To our knowledge, this is the first time *Prochlorococcus* has been challenged with simulated H_2_O_2_ accumulation over the day while in co-culture with helpers, and future studies may provide additional insight into the roles that each helper may play under these dynamic situations.

Experiments in this and prior studies from our group (22, 41, 55) examined *Prochlorococcus* growth during chronic, though sometimes dynamic, exposure to H_2_O_2_. To assess acute H_2_O_2_ exposures, we exploited the ability of pyruvate to eliminate H_2_O_2_ within minutes after addition. We observed that exposure windows of 12 hours or more resulted in significant loss of *Prochlorococcus* viability, as quantified by loss of detectable cells via flow cytometry. This has implications for rainfall events that provide temporary elevated concentrations of peroxide in the surface seawater (13, 14, 16) as well as diel periodicities in maximal peroxide concentration (17, 19, 23, 53, 54). Notably, these losses in viability were not apparent immediately but were observed 48 hours after the initial exposure to H_2_O_2_. These results suggest that peroxide-killed cells may retain autofluorescence via chlorophyll for several days before it is eventually lost. Future studies should be aimed at a deeper investigation of the intracellular dynamics during peroxide-mediated mortality.

### Contribution of heterotrophs and photoautotrophs to the microbial sink

**Fig 6:**
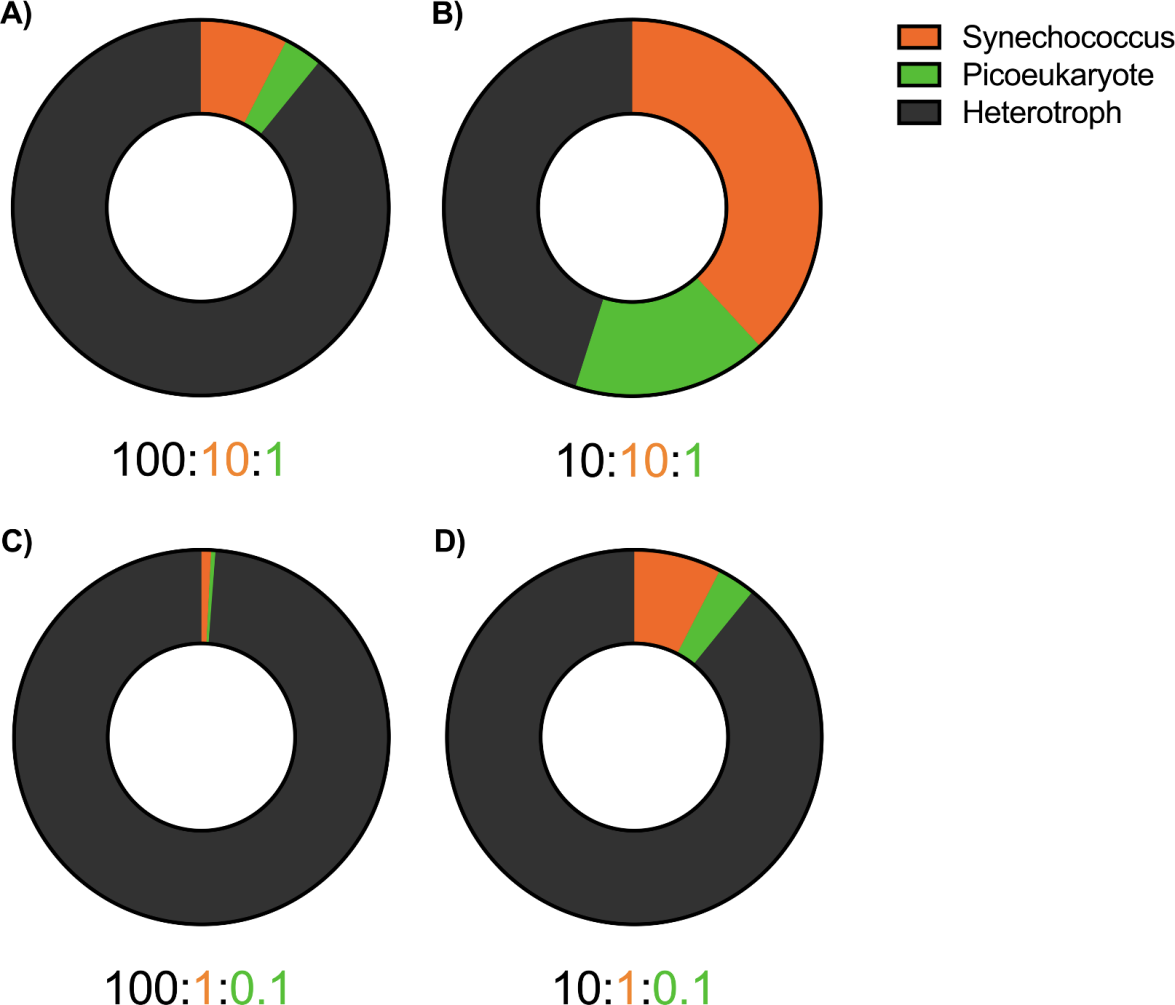
Community H_2_O_2_ degradation. Relative contributions to the microbial sink (nM day^−1^ cell^−1^) by *Synechococcus*, picoeukaryotic phytoplankton (*Micromonas* and *Ostreococcus*), and heterotrophic bacteria (*Alteromonas*), assuming environmental abundances of *Synechococcus* at 10^4^, picoeukaryotes at 10^3^, and *Alteromonas* at (**A**) 10^5^ or (**B**) 10^4^ cells mL^−1^, followed by environmental abundances of *Synechococcus* at 10^3^, picoeukaryotes at 10^2^, and *Alteromonas* at (**C**) 10^5^ or (**D**) 10^4^ cells mL^−1^. Individual decay rates were determined for instantaneous addition of 750 nM: *Synechococcus* at 0.002, picoeukaryotes at 0.008, and heterotrophs at 0.00236 nM day^−1^ cell^−1^.

As an initial effort to contextualize the contributions of co-occurring phytoplankton to the H_2_O_2_-degrading microbial sink from which *Prochlorococcus* benefits, we “assembled” communities of *Synechococcus*, picoeukaryotes, and heterotrophs at ecologically relevant concentrations and applied their per cell degradation rates (calculated by linear regression) to their appropriate population sizes. Results for exposures to instantaneous 750 nM H_2_O_2_ additions (Fig. 6) were nearly identical to those for 300 nM additions (Fig. S7).

In the first case, we considered the *Alteromonas* strain as a proxy for all heterotrophs in the open ocean, catalase positive or not, and set their concentration to 10^5^ cells mL^−1^ (56–58), while placing *Synechococcus* (10^4^ cells mL^−1^), and picoeukaryotes (10^3^ cells mL^−1^) at their reported cell abundances (33, 34). In this community model, it was clear that the heterotrophs were responsible for the vast majority of H_2_O_2_ decay (Fig. 6A). This may be a significant overestimation of the heterotrophic contribution to the microbial sink, as some of the more abundant lineages such as SAR11 have genotypes that lack catalase (39).

In the second case, we set the *Alteromonas* concentration 10-fold lower to reflect a more conservative estimate of the abundance of this genus (59–61). In this case, the combined activity of the photosynthetic *Synechococcus* + picoeukaryotes accounted for over half of the H_2_O_2_ degradation (Fig. 6B). A comparison of the two cases highlights the importance of understanding the abundance of catalase-positive genotypes among the total heterotrophic community; such understanding should be targeted in future studies of open ocean community composition. Both cases, but especially case two, predict the production of catalase-peroxidase or ascorbate peroxidase for *Synechococcus* and picoeukaryotic phytoplankton, respectively, providing a net positive interaction for *Prochlorococcus* and a necessary but twofold detriment for the others: production consumes internal nutrient stores, requiring higher cell quotas, and H_2_O_2_ degradation promotes the survival of their numerically dominant competitor, *Prochlorococcus*.

Like the uncertainty involving catalase-positive heterotrophs, we note several caveats to our phytoplankton estimates. Several strains of marine *Synechococcus* lack catalase (39, 62) and presumably would not degrade peroxide nearly as well as catalase-positive strains. Our calculations assume 100% of the *Synechococcus* cells are catalase positive, but we acknowledge this is likely to be an overestimate and that the actual contributions of the diverse *Synechococcus* populations in the open ocean (63–66) are probably lower. Additionally, while we have generated empirical data for several isolates of *Micromonas* and *Ostreococcus*, reports indicate that the dominant picoeukaryotes have yet to be cultured (47, 67, 68), and their contribution to the microbial sink is currently unknown. If, as we modeled for the heterotrophic community (Fig. 6A and B and Fig. S7A and B), we assume only a fraction of the phytoplankton populations provides catalase activity (provisionally set to 10%), we see that the vast majority of protection comes from the heterotrophs (Fig. 6C and D and Fig. S7C and D). Despite these uncertainties, our empirical results provide initial upper and lower constraints on the phytoplankton contribution to the “helper” microbial sink in the open ocean.

### Evolutionary significance

The Black Queen Hypothesis (BQH) describes evolutionary outcomes where a beneficial change in fitness or physiological costs occurs by loss of leaky functions that can be provided by other community members, such as nutrient acquisition, polymer degradation, or environmental detoxification (39, 69, 70). The BQH was conceived to describe the evolutionary loss of catalase in *Prochlorococcus*, with the assignment of leaky “helper” given to heterotrophic bacteria. With the outcome of this work, we pose a follow-up question: did co-occurring phytoplankton contribute to the evolutionary loss of catalase in *Prochlorococcus*? While impossible to answer, we suggest that the propensity of phytoplankton to mitigate peroxide damage via a leaky degradation process, coupled with their significant abundances in the present-day open ocean, suggests that they contributed indirectly to the emergence of *Prochlorococcus* as the numerically dominant member of the phytoplankton community.

### Conclusions

Here, we determined that open ocean populations of *Synechococcus*, *Micromonas*, and *Ostreococcus* are all capable of protecting *Prochlorococcus* from exposure to lethal concentrations of H_2_O_2_ and likely contribute significantly to the degradation activity of the entire microbial community. These results highlight the complexity of inter-trophic interactions in the open ocean as co-occurring phytoplankton can compete with but also protect their competitors.

## MATERIALS AND METHODS

### Strains and culturing

Axenic cultures of strains of picocyanobacteria *Prochlorococcus* (MIT9215) and *Synechococcus* (WH7803 and CC9605), picoeukaryotic phytoplankton *Micromonas* (RCC299 and CCMP1545), and *Ostreococcus* (CCMP2972A), and marine heterotroph *Alteromonas macleodii* (EZ55) were used in this study. All cyanobacterial stock cultures were maintained in an artificial seawater medium, AMP-A, identical to AMP-J (41) except that the basal salts medium is autoclaved rather than filter sterilized. Picoeukaryote stock cultures were maintained, and all experiments were performed using an AMP-A derivative, AMP-PE (for Pico-Eukaryotes, this study), which allowed for efficient and consistent growth of all photosynthetic microbes in mono- and coculture. This medium has an identical recipe and preparation as AMP-A except for the following alterations: 10× addition of trace metal working stock, 1.06e^−4^ M silica, 2.96e^−7^ M thiamine, 2.05e^−9^ M biotin, and 3.69e^−10^ M cyanocobalamin. Stocks of these nutrients were filter sterilized and added after sterilization of the base saltwater medium. Axenic heterotrophic bacteria *Alteromonas macleodii* strain EZ55 (40) was inoculated from cryo-preserved stocks prior to each experiment (−80°C in YTSS + 10% glycerol) into 5 mL YTSS (71) and incubated shaking at 140 RPM at 24°C overnight. Before inoculation into experimental cultures, the heterotroph was washed three times in 1.5 mL microcentrifuge tubes by centrifugation at 8,000 RPM for 2 minutes in a tabletop microcentrifuge and resuspension in 1 mL AMP-A. All experiments were carried out in duplicate or triplicate at 24°C in Percival I36VLX incubators (Percival, Boone, IA) that allowed for gradual increase and decrease of cool white light to simulate sunrise and sunset with peak midday light intensity of 150 µmol quanta m^−2^s^−1^ on a 14 h:10 h light:dark cycle (72). For experiments involving heat-killed cells, cultures of helper phytoplankton were quantified by flow cytometry then incubated at 95°C for 20 minutes and added to the medium as performed for their live cell counterparts. Notably, the heat killed cells retained forward scatter and autofluorescence properties as assessed by flow cytometry. Purity tests to determine the axenicity of cyanobacteria and picoeukaryote stock and experimental cultures were routinely performed as previously described (40). Data for the decay kinetics of *Alteromonas* monocultures used to generate Fig. 6 and Fig. S6 are unpublished (D. K. McCullough, E. C. Bowden, B. C. Calfee, M. A. Gilchrist, E. R. Zinser, and D. Talmy).

For the H_2_O_2_ variable exposure time and heat-killed phytoplankton experiments, cells were grown in AMP-PE at 22°C with a static (non-ramping) light intensity of 100 µmol quanta m^−2^s^−1^ on a 14 h:10 h light:dark cycle. Variable exposure time experiments were started 4 hours after the onset of the light period when cells from mid-exponential cultures were inoculated into AMP-PE supplemented with varying concentrations of H_2_O_2_. Sodium pyruvate is a rapid (i.e., minutes) and effective means of eliminating H_2_O_2_ from the medium (73–75), and these properties allowed us to pulse cultures of *Prochlorococcus* with hydrogen peroxide for defined periods of time, followed by immediate removal by pyruvate addition. To rapidly eliminate exogenous H_2_O_2_ after desired exposure times, 500 µM sodium pyruvate was added to the medium. The addition of this concentration of pyruvate depleted all concentrations of H_2_O_2_ within a few minutes (data not shown).

### Cell abundance quantification

Abundances of cyanobacteria were quantified by flow cytometry using a CytoFLEX S flow cytometer (Beckman Coulter, Brea, CA) with populations of *Prochlorococcus* and *Synechococcus* differentiated in cocultures by their red (675 nm) and red/yellow (675 nm/578 nm) fluorescence, respectively (40, 76). Picoeukaryotes were quantified by red (675 nm) and far red (770 nm) fluorescence. Detection of red and yellow fluorescence was achieved after excitation with a blue (488 nm) laser, while detection of far-red fluorescence required excitation by a yellow (565 nm) laser. Quantification of *Prochlorococcus* in coculture was achieved by observing events determined by red fluorescence after events that corresponded to the fluorescence properties of either *Synechococcus* (red/yellow) or picoeukaryotes (red/far red) were removed from abundance calculation. Heterotrophs in coculture experiments were quantified by viable counting with serial dilutions on YTSS 1.5% agar plates incubated at 24°C.

### Hydrogen peroxide quantification and addition

The concentration of HOOH in the medium and cultures was measured on an Orion L Microplate Luminometer (Titertek Instruments Inc., Berthold Detection Systems, Pforzheim, Germany) using an acridinium ester (Cayman Chemical Company, Ann Arbor, MI) chemiluminescence method (22). Concentrations in cultures were adjusted via both instantaneous and incremental (during 14 hour light period) addition to achieve specific exposure conditions, as described in the figure legends. Depending on the residual H_2_O_2_ in prepared AMP-PE media, incremental H_2_O_2_ ramping was achieved by consecutive 100, 100, 100, 175, and 200 nM additions at 0, 3, 6, 9, and 12 hours, respectively.

## Data Availability

Prochlorococcus and Synechococcus cell density and hydrogen peroxide concentration data are available through the Biological and Chemical Oceanography Data Management Office (BCO-DMO, Zinser et al. 2023) at https://doi.org/10.26008/1912/bco-dmo.913181.1 and the Github repository “Rival-phytoplankton-contribute-to-the-cross-protection-of-Prochlorococcus-from-oxidative-stress" at https://github.com/bcalfee/Rival-phytoplankton-contribute-to-the-cross-protection-of-Prochlorococcus-from-oxidative-stress.git.

## SUPPLEMENTAL MATERIAL

Supplemental material aem.01128-24-s0001.docx Table S1; Figures S1 to S7.

ASM does not own the copyrights to Supplemental Material that may be linked to, or accessed through, an article. The authors have granted ASM a non-exclusive, world-wide license to publish the Supplemental Material files. Please contact the corresponding author directly for reuse.
