# Supplementary material for: Rival phytoplankton contribute to the cross protection of Prochlorococcus from oxidative stress
Source: Appl Environ Microbiol. 2025 Apr 10;91(5):e01128-24. doi: 10.1128/aem.01128-24 (PMC12094017; doi:10.1128/aem.01128-24)
Supplement: Supplemental material — Table S1; Figures S1 to S7. [file aem.01128-24-s0001.docx]

**Supplemental Material**


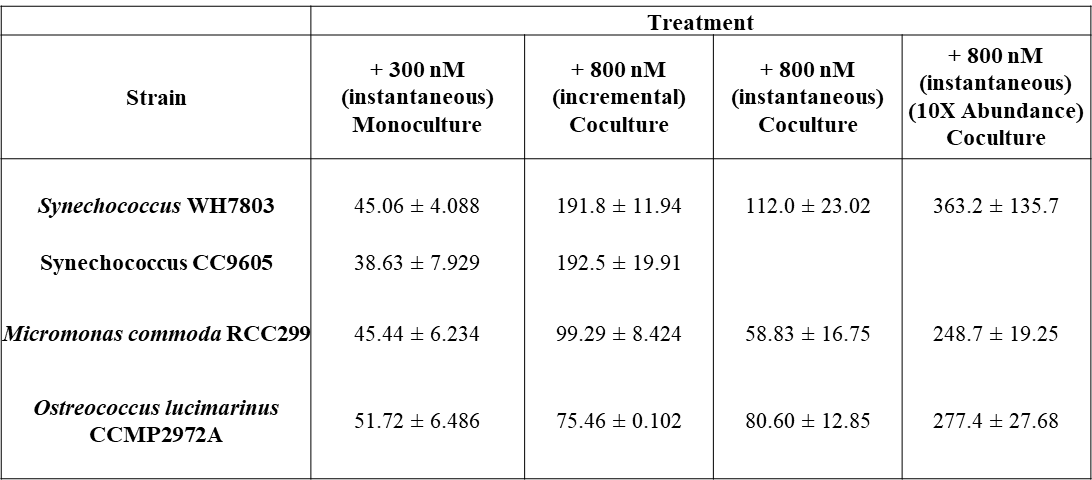


**Table S1. H_2_O_2_ Degradation Rates:** Degradation rates (nM day^-1^) of H_2_O_2_ by *Synechococcus*, *Micromonas*, and *Ostreococcus* strains when in coculture with *Prochlorococcus*, exposed to either instantaneous or incremental addition of H_2_O_2_. Rates were calculated as the slope of a linear regression using at least three time points.





**Fig. S1. Survival of *Synechococcus* during simulated rainfall:** Growth of *Synechococcus* strains (A) WH7803 and (B) CC9605 in monoculture and coculture with *Prochlorococcus* MIT9215 in AMP-PE artificial seawater medium after addition of 0 nM or 300 nM H_2_O_2_. Concentrations of H_2_O_2_ quantified for monocultures of all strains and an abiotic control with addition of (C) 0 nM or (D) 300 nM H_2_O_2_ (n=2).

**
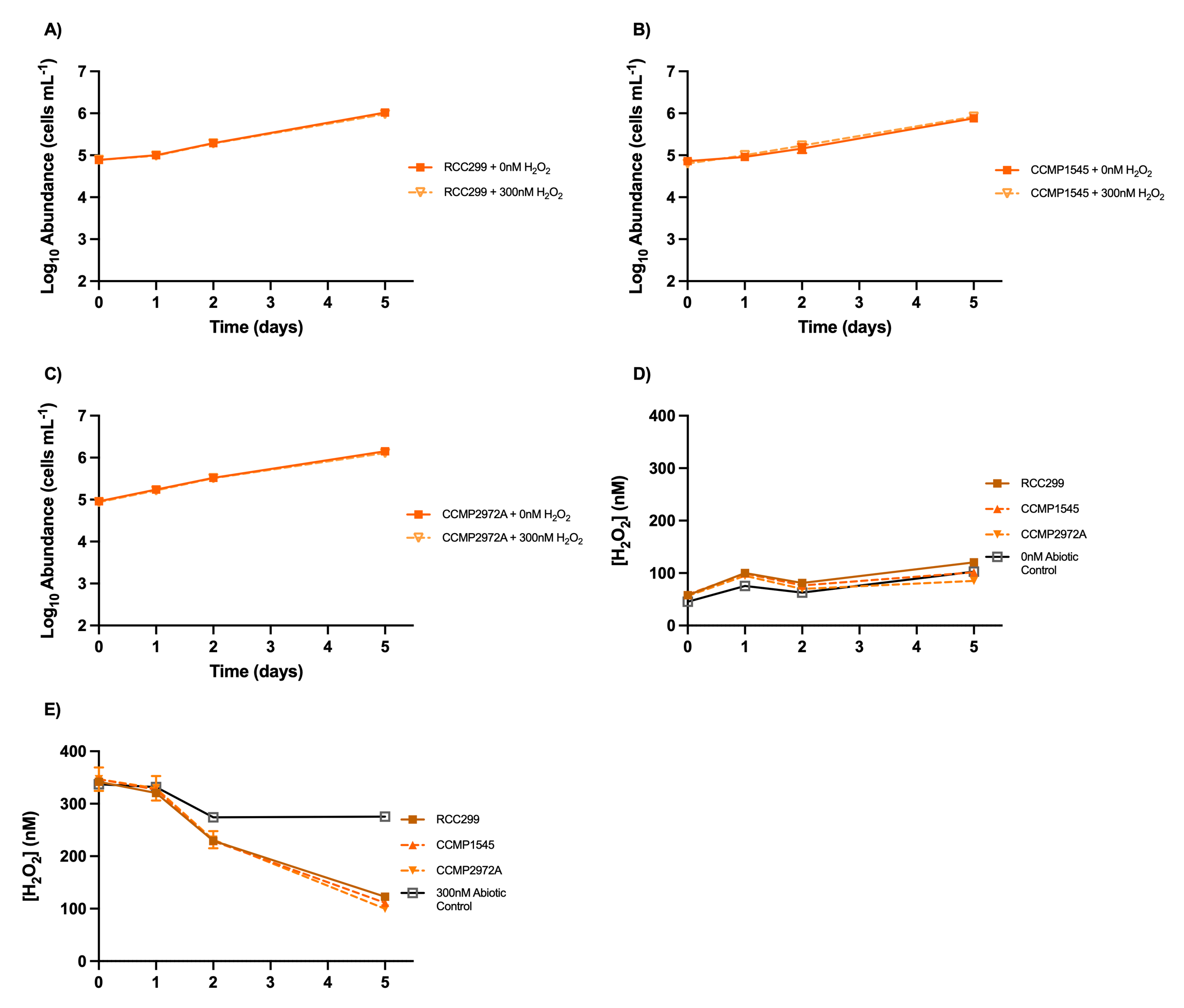
**

**Fig. S2. Survival of picoeukaryotes during simulated rainfall:** Growth of (A) *Micromonas commoda* strain RCC299, (B) *Micromonas pusilla* strain CCMP1545, and (C) *Ostreococcus lucimarinus* strain CCMP2972A in monoculture in AMP-PE artificial seawater medium after addition of 0 nM or 300 nM H_2_O_2_. Concentrations of H_2_O_2_ quantified for monocultures of all strains and an abiotic control with addition of (D) 0 nM or (E) 300 nM H_2_O_2_ (n=2).





**Fig. S3. Survival of *Prochlorococcus* after Instantaneous Addition of 750 nM H_2_O_2_:** Growth of *Prochlorococcus* strain MIT9215 (abbreviated Pro in figure key) in mono- and coculture with (A) *Synechococcus* strain WH7803, (B) *Micromonas commoda* strain RCC299, (C) *Ostreococcus lucimarinus* strain CCMP2972A, or (all panels) *Alteromonas macleodii* strain EZ55 in AMP-PE artificial seawater medium exposed to an instantaneous addition of ~750 nM H_2_O_2_ (n=2). The initial abundance of photosynthetic helpers was either 1x or 10x ([10x]) their ecologically relevant abundance. Within each legend, parentheses represent the addition to a particular strain / treatment.


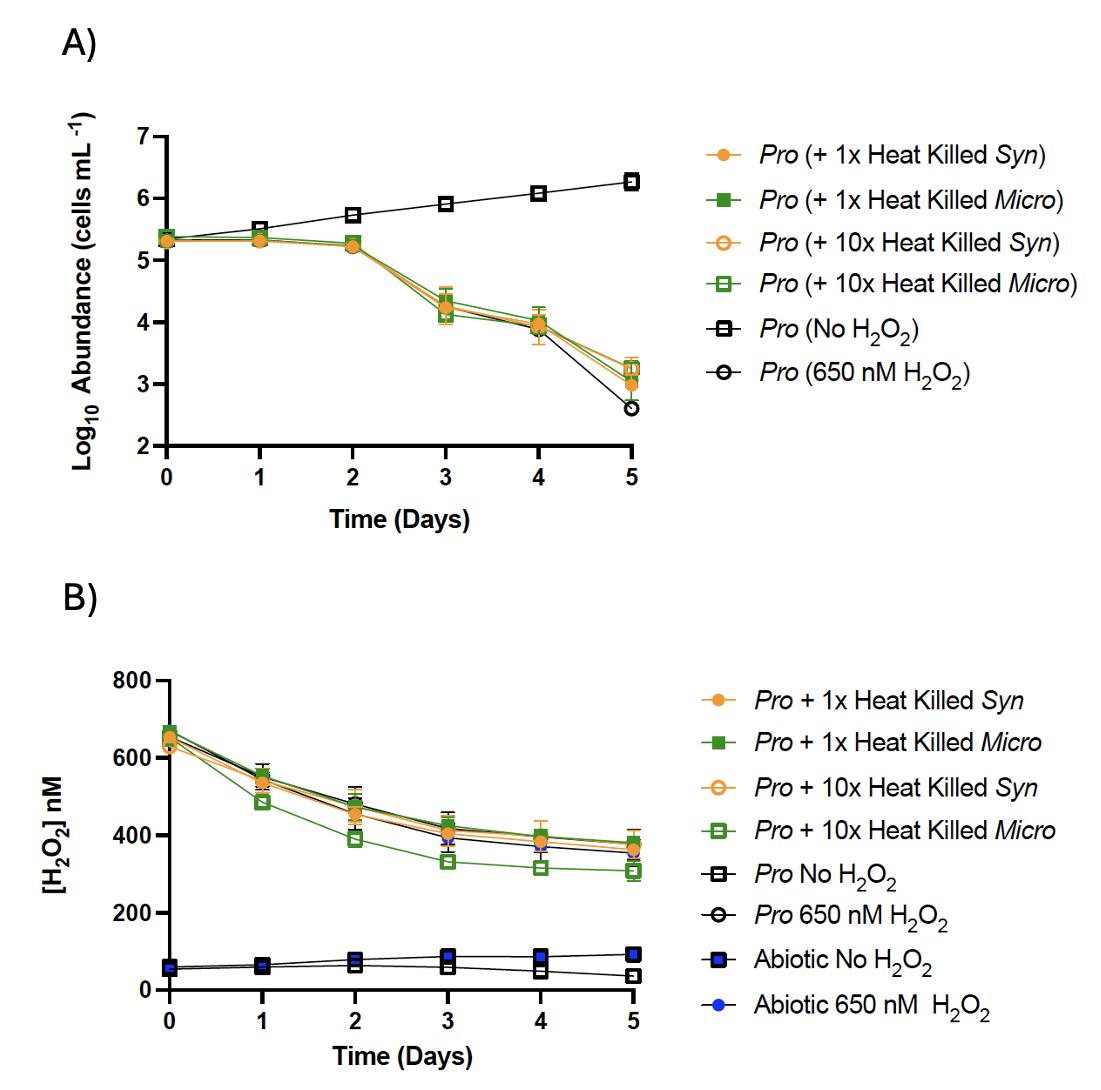


**Fig. S4. Growth of *Prochlorococcus* with Heat-Killed *Synechococcus* and *Micromonas* after Instantaneous Addition of 650 nM** **H_2_O_2_:** (A) Growth of *Prochlorococcus* strain MIT9215 (abbreviated Pro in figure key) in mono- and coculture with heat-killed *Synechococcus* strain WH7803 and *Micromonas commoda* strain RCC299 in AMP-PE artificial seawater medium exposed to an instantaneous addition of ~650 nM H_2_O_2_. (B) Daily H_2_O_2_ concentrations from all coculture treatments (n=3). The initial abundance of photosynthetic helpers was either 1x or 10x their ecologically relevant abundance. Within each legend, parentheses represent the addition to a particular strain / treatment.





**Fig. S5. Growth of *Synechococcus* and Picoeukaryotes After Simulated Photochemical Production of H_2_O_2_:** Growth of (A) *Synechococcus* strains WH7803 and CC9605, (B) *Micromonas commoda* strain RCC299 and *Ostreococcus lucimarinus* strain CCMP2972A in coculture with *Prochlorococcus* strain MIT9215 in AMP-PE artificial seawater medium exposed to an incremental addition of 800 nM (*Synechococcus*) or ~650 nM (picoeukaryotes) H_2_O_2_ over the course of the daylight portion of a single diel (n=3). Within each legend, parentheses represent the addition to a particular strain / treatment.

**
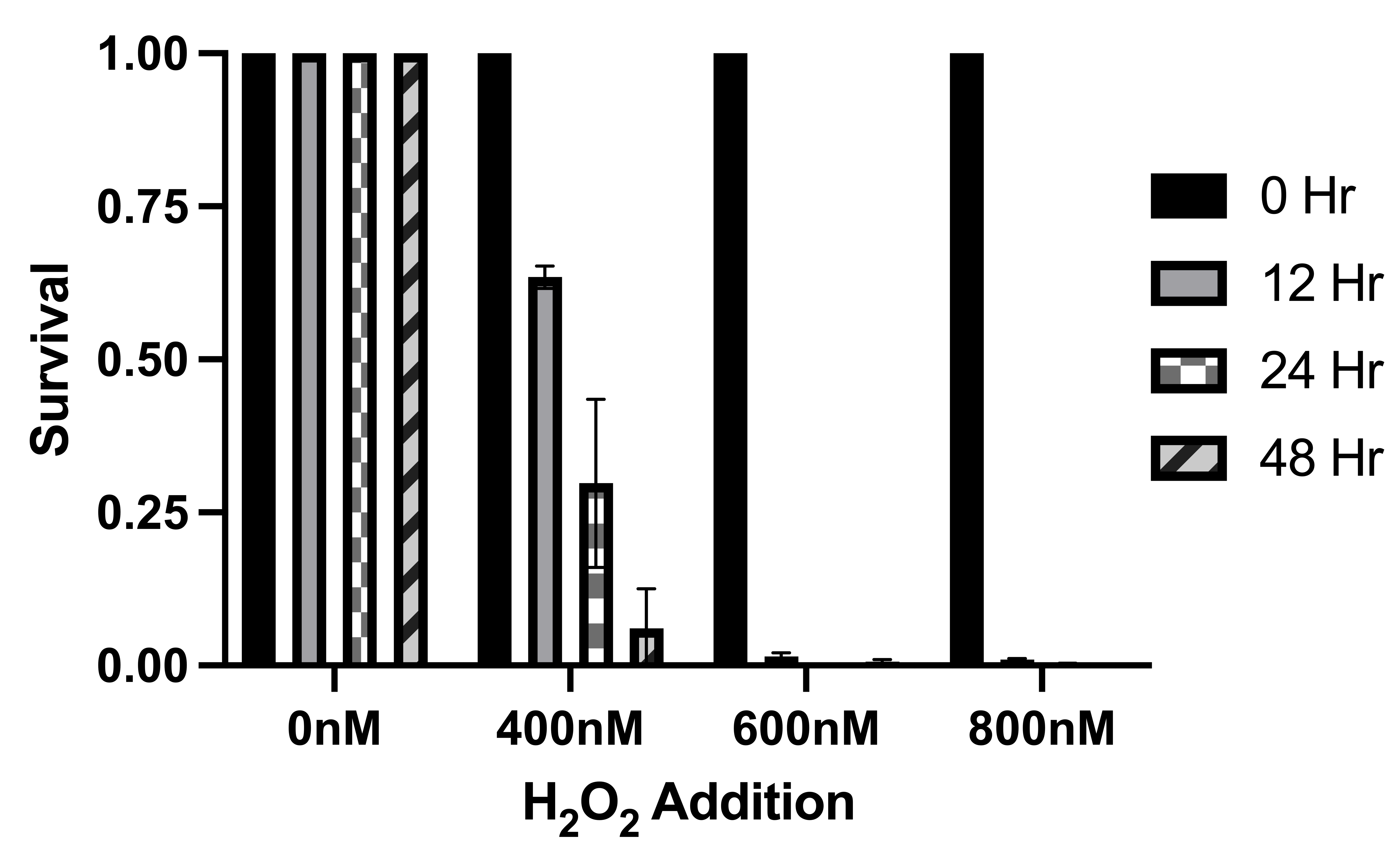
**

**Fig. S6. *Prochlorococcus* abundance after varying exposures to H_2_O_2_:** Percent survival of *Prochlorococcus* strain MIT9215 in monoculture in AMP-PE artificial seawater medium supplemented with 0, 400, 600, or 800 nM H_2_O_2_. H_2_O_2_ was completely removed after 0, 12, 24, or 48 hours of exposure by addition of 500 μM sodium pyruvate. Survival reported for hour 120 as the fraction of cell counts relative to the 0 hr H_2_O_2_ exposure. Error bars represent one standard deviation of the geometric mean (n=3).

**
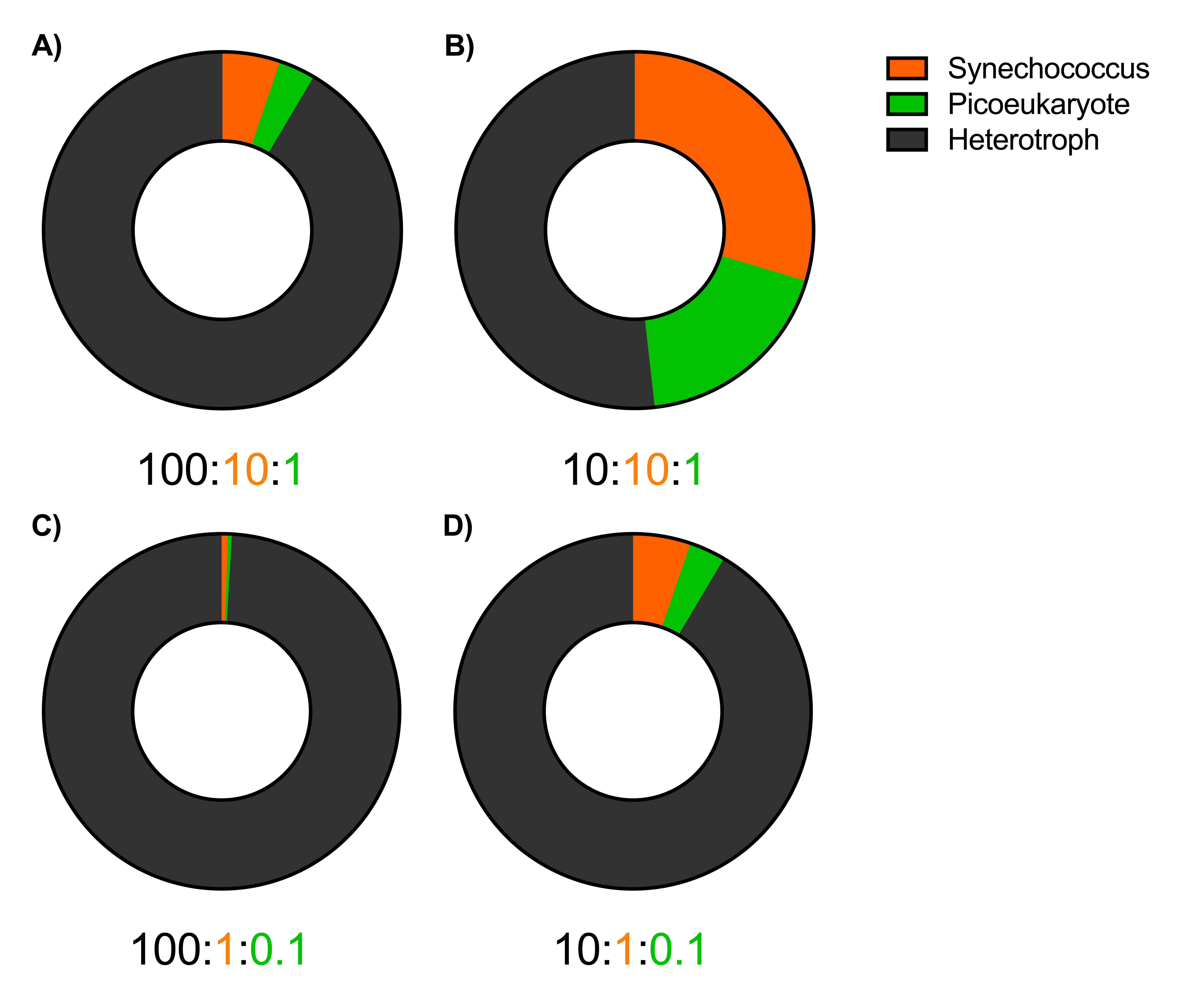
**

**Fig. S7.** **Community H_2_O_2_ Degradation:** Relative contributions to the microbial sink (nM day^-1^ cell^-1^) by *Synechococcus*, picoeukaryotic phytoplankton (*Micromonas* and *Ostreococcus*), and heterotrophic bacteria (*Alteromonas*), assuming environmental abundances of *Synechococcus* at 10^4^, picoeukaryotes at 10^3^, and *Alteromonas* at (A) 10^5^ or (B) 10^4^ cells ml^-1^, followed by environmental abundances of *Synechococcus* at 10^3^, picoeukaryotes at 10^2^, and *Alteromonas* at (C) 10^5^ or (D) 10^4^ cells ml^-1^. Individual decay rates were determined for instantaneous addition of 300 nM: *Synechococcus* at 0.0005, picoeukaryotes at 0.0032, and heterotrophs at 0.0009 nM day^-1^ cell^-1^.
